# Case Report: Clinicopathological and Genetic Features of IDH-Mutant Brainstem Glioma in Adults: Report of Five Cases

**DOI:** 10.3389/pore.2022.1610408

**Published:** 2022-08-04

**Authors:** Jiangfen Zhou, Mingyao Lai, Yang Ni, Shaoqun Li, Junjie Zhen, Furong Du, Xing Zhang, Chao Song, Linbo Cai

**Affiliations:** ^1^ Department of Neuro-Oncology, Guangdong Sanjiu Brain Hospital, Guangzhou, China; ^2^ State Key Laboratory of Translational Medicine and Innovative Drug Development, Jiangsu Simcere Diagnostics Co., Ltd., Nanjing, China

**Keywords:** prognosis, IDH1 mutation, molecular features, temozolomide, brainstem glioma

## Abstract

Adult brainstem gliomas are rare central nervous system tumors that represent a heterogeneous group of tumors. Somatic *IDH* mutations are uncommon in adult brainstem gliomas and there are few relevant clinical studies. Here, we reported five patients with *IDH1* mutations associated with brainstem gliomas, including four cases of *IDH1* R132H mutations and one case of R132G mutation. All patients were treated with focal intensity-modulated radiation therapy (IMRT) with concurrent temozolomide (TMZ). One patient died, one relapsed, and three survived to date. All these cases carried a pathogenic variant of *TP53*, among whom 1 harbored *ATRX* mutation and 1 had *H3K27M* mutation. Moreover, we also found some genes related to a worse prognosis, such as *CDK4/6* amplification. These findings demonstrate that the specific characteristics of *IDH*-mutant brainstem gliomas should be considered in diagnostic workflows to make therapeutic regimens and improve the prognosis.

## Introduction

Brainstem gliomas are relatively rare in the central nervous system, only representing 1–2% of all adult brain tumors [1]. Adult brainstem gliomas represent a heterogeneous group of tumors arising from the midbrain (12–15%), pons (60–63%), or medulla oblongata (25%) [2]. Based on clinicopathologic and magnetic resonance imaging (MRI) characteristics, they are classified into diffuse, intrinsic focal, extrinsic focal, and cervicomedullary types [3]. In contrast to pediatric gliomas, isocitrate dehydrogenase (IDH) mutations are more frequent in adult low-grade glioma [4]. However, *IDH*-mutant gliomas arising from the brainstem are exceedingly rare in adults and children. In this study, we described the clinicopathological and molecular characteristics of the five adult patients with *IDH*-mutant brainstem gliomas (Table 1 and Supplementary Table S1) in detail.

**TABLE 1:** Clinocopathological and molecular characteristics of the five adults with brainstem gliomas.

Case	Histology	Age	Sex	Tumor location	Clinical manifestations	Molecular findings	Follow-up period
#1	Diffuse astrocytoma	34	Male	Brainstem	Dizziness, diplopia, hearing loss in the right ear, dextral numbness	*IDH1* R132H, *TP53* A88P, *ATRX* R2197H, *CDK4* amplification, *ISR2* amplification	3 months
#2	Low-grade glioma	49	Male	Brainstem	Limb weakness, dysarthria, ataxia	*IDH1* R132H, *TP53* S261V, *KRAS* (G12V), *H3F3A* amplification	17 months
#3	Diffuse astrocytoma	29	Male	Brainstem	Episodic dizziness, diplopia, facial palsy	*IDH1* R132H, *TP53* C238F, *PTEN* deletion, *KMT2A* (exon1)*-CBL* (exon2-1) fusion, segment deletion (1q, 4q, 5q, 7p, 10q, 11q, 12q, 13q)	9 days
#4	Diffuse astrocytoma	52	Female	Brainstem	Dizziness, ataxia, mild headache	*IDH1* R13*2H, HIST1H3B *K27M, *TP53* R267P, *CDK6* amplification, chromosomal amplification (3q11.1-q22.1, 3p22.1-p21.31) and deletion (3q11.1-q22.1)	13 months
#5	Diffuse astrocytoma	32	Male	Brainstem	Weakness in the right limb	*IDH1* R132G, *TP53* R273H, *CDK4* amplification, *BRCA2* K2729N, chromosomal deletion (21q11.2-q22.3,1q23.3-q44)	28 months

## Case Description

**FIGURE 1:**
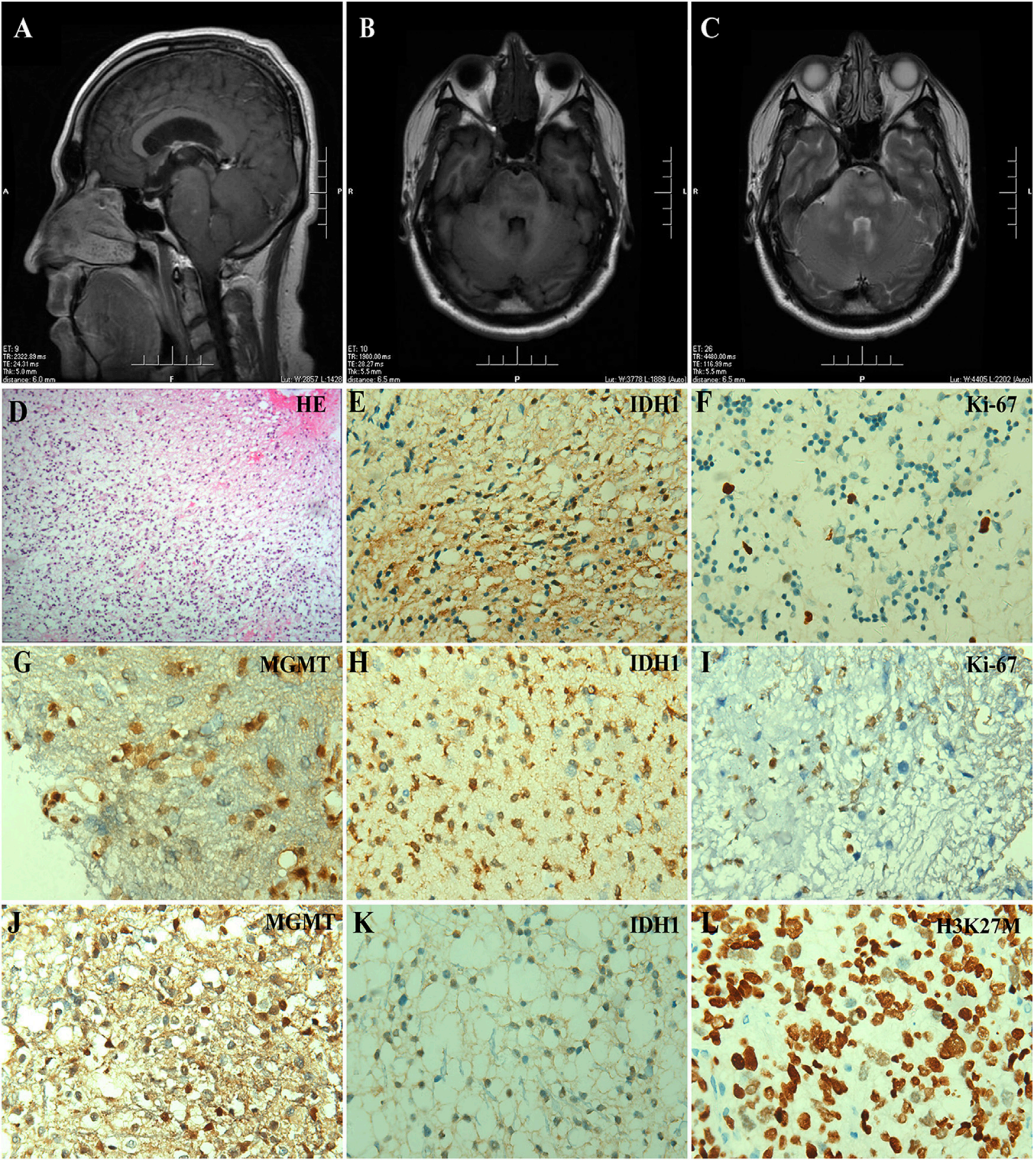
*IDH*-mutant brainstem gliomas in adult patients. **(A–C)** Preoperative MRI scanning of Case #1, including a plain scan of T1-weighted **(A)** and T2-weighted **(B)** images in axial views and a contrast-enhanced scan of T1-weighted images in sagittal view **(C)**. The arrowhead indicated the lesion in the brainstem. **(D–F)** Histologic and immunohistochemical features of the tumor from Case #1. **(D)** HE staining, **(E)** IDH1 R132H, and **(F)** Ki-67 immunostaining (×400). **(G–I)** Immunohistochemical staining of the tumor from Case #2, including **(G)** MGMT, **(H)** IDH1 R132H, and **(I)** Ki-67 (×400). **(J–L)** Immunohistochemical staining of the tumor from Case #4, including **(J)** MGMT, **(K)** IDH1 R132H, and **(L)** H3K27M (×400).

Case #1 was a 34-year-old man who developed dizziness, diplopia, hearing loss in the right ear, and dextral numbness in December 2018. The brain MRI indicated obvious swelling and abnormal signals in the cervical medullary segment, brainstem, and bilateral bridge arms in May 2019, considering the possibility of high-grade glioma (Figures 1A–C), thus the brainstem space-occupying excision was performed. Histology revealed a diffuse growth in the tumor with moderately increased cell density (Figure 1D). By immunohistochemistry (IHC), IDH1-positive tumor cells (Figure 1E) and Ki-67 of 5% were observed (Figure 1F). Fluorescence *in situ* hybridization (FISH) analysis showed that chromosomes 1p and 19q were not deleted, but *IDH1* R132H mutation was confirmed by Sanger sequencing. The targeted next-generation sequencing (NGS) using a panel of 131 genes and 4 chromosomes [5] (131 + 4 panel, Supplementary Table S2) further identified mutations in *IDH1*, *TP53,* and *ATRX*, *CDK4* amplification, and *IRS2* amplification. In combination with histopathology and molecular analysis, the patient was diagnosed with astrocytoma, *IDH*-mutant WHO grade II. He was given anti-epileptic therapies due to pre- and post-operative epileptic seizures and got better after focal intensity-modulated radiation therapy (IMRT, DT 54Gy/27f) with concurrent temozolomide (TMZ, 120 mg). In March 2021, the tumor relapsed due to the presence of enlarged lesions in the callosum-septum pellucidum area and multiple lesions as before, and then concurrent radiochemotherapy and symptomatic treatment were applied. Approximately 20 days after treatment, the patient was discharged from the hospital and continued to receive radiochemotherapy as an outpatient.

Case #2 was a 49-year-old man with limb weakness, dysarthria, ataxia, and sometimes dizziness. Brain MRI and magnetic resonance spectrum (MRS) showed a brainstem lesion, thus the brainstem biopsy was conducted. Low-grade glioma was considered. Tumor cells were positive for O-6-methylguanine-DNA methyltransferase (MGMT) and IDH1, with a proliferation activity of 2% for Ki-67 (Figures 1G–I). *IDH1*, *TP53,* and *KRAS* mutations and *H3F3A* amplification were detected using the targeted 131 + 4 panel. The patient was treated with focal IMRT (DT 54Gy/27f) with concurrent TMZ (75 mg/m^2^) and TMZ maintenance therapy after hospital discharge. The patient survived, with weakness in the lower limbs.

Case #3 was a 29-year-old man with episodic dizziness, diplopia, and facial palsy for 1 month. He received a robot-guided biopsy due to brain MRI findings of a space-occupying lesion between the brainstem and the right cerebellar hemisphere. Microscopically, the tumor showed diffuse invasive growth with moderately increased cell density. Tumor cells were positive for MGMT and IDH1, with a proliferation activity of 4% for Ki-67. Through the targeted 131 + 4 panel, it was found that *IDH1* and *TP53* mutations, *PTEN* deletion, *KMT2A*-*CBL* fusion, and segment deletion of the chromosome. Based on these findings, the patient was diagnosed with astrocytoma, *IDH*-mutant WHO grade II, and was treated with focal IMRT (DT 54Gy/30f) with concurrent TMZ (75 mg/m^2^). Three weeks later, the peritumoral edema improved. However, 2 months after treatment, the computed tomography (CT) indicated a slowly increasing brainstem hemorrhage. Family members refused for the patient to be transferred to the intensive care unit, and he was dead 9 days later from brainstem hemorrhage.

Case #4 was a 52-year-old woman experiencing dizziness, ataxia, and mild headache in 2019. Brain CT, MRI, and MRS all suggested space-occupying neoplasms in the brainstem in November 2020. Under general anesthesia, brainstem glioma excision, decompressive craniectomy, and extra ventricular drainage were performed. Tumor cells were positive for MGMT, IDH1, and H3K27M (Figures 1J–L), with Ki-67 of 7%. Interestingly, the targeted 131 + 4 panel revealed mutations in *IDH1*, *H3K27M,* and *TP53*, *CDK6* amplification, as well as chromosomal amplification (3q11.1-q22.1, 3p22.1-p21.31) and deletion (3q11.1-q22.1). She was diagnosed with astrocytoma, *IDH1*-mutant, *H3K27M*-mutant WHO grade II. Due to economic factors, the patient refused chemotherapy and received conformal radiotherapy. She was discharged from the hospital for personal reasons and has survived to date.

Case #5 was a 32-year-old man with weakness in the right limb in December 2018. Brain MRI in May 2019 indicated a space-occupying lesion in the medulla oblongata, considered a low-grade glioma. After the brainstem glioma excision, dura mater reparation, and cranioplasty, the patient was diagnosed with astrocytoma, *IDH*-mutant WHO grade II based on histopathology and molecular analysis which showed mutations in *IDH1* and *TP53*, *CDK4* amplification, *BRCA2*, and chromosomal deletion (21q11.2-q22.3,1q23.3-q44) using the targeted 131 + 4 panel. Focal IMRT (DT 40Gy/20f) with concurrent TMZ (120 mg) was used. About 1 month after treatment, the patient’s symptoms improved, and he left the hospital following the fourth cycle of TMZ chemotherapy (340 mg). According to the doctor’s advice, the subsequent fifth and sixth cycles of TMZ chemotherapy (340 mg) were used. The disease was stable 2 years after treatment.

## Discussion

*IDH* mutations are uncommon in brainstem gliomas. The majority (90%) of astrocytomas and oligodendrogliomas carry a canonical *IDH1* R132H mutation [6], which can be detected by IDH1 (R132H), while other mutations should be detected through the sequencing involving *IDH1* and *IDH2* genes. In our report, the tumor cells from four cases showed positive for *IDH1* (R132H), which were confirmed by sequencing techniques, and a noncanonical *IDH1* R132G mutation was identified in Case #5 by Sanger sequencing. All these cases carried a pathogenic variant of *TP53*, further suggesting the importance of *TP53* mutations in astrocytoma. Previous studies indicated that the *TP53* rs4968187 variant was associated with the risk of developing astrocytoma, and the *TP53* rs78378222 variant was significantly connected with a higher risk of glioblastoma [7, 8].

By analyzing the genetic alterations in diffuse low‐grade gliomas, Aoki et al. found that altered RB pathway genes including *CDKN2A* and *CDK4* could be independent predictors of poor survival in *IDH*-mutant astrocytomas [9]. Among these five patients, however, no *CDKN2A/B* deletion was identified, but *CDK4*/*6* amplification was found in Cases #1, #4, and #5. Selective inhibition of CDK4/6 was reported to suppress the tumor growth and may enhance the sensitivity of glioma cells to TMZ, suggesting that CDK4/6 inhibition may be a favorable treatment strategy for glioma and overcome TMZ resistance [10]. Currently, abemaciclib and ribociclib, two kinds of CDK4/6 inhibitors, are being explored in phase I trials for pediatric and adult patients with glioblastoma, although palbociclib, a dual CDK4/6 inhibitor, is ineffective in the treatment of recurrent glioblastoma [11–13].

In our report, the tumor cells of three patients were positive for MGMT, whose promoter methylation (Supplementary Figure S1) is considered a strong prognostic biomarker in pediatric and adult glioblastoma patients treated with TMZ [14, 15]. In Case #4, a *H3K27M* mutation was identified. This mutation could lead to a unique relatively homogenous phenotype with key epigenetic, genetic, and clinical significance. Notably, mutations in *H3K27M* and *IDH1* may be mutually exclusive. Compared with other *IDH*-mutant infratentorial gliomas, two patients with noncanonical *IDH1* R132C and *H3K27M* mutations showed shorter overall survival [16].

**FIGURE 2:**
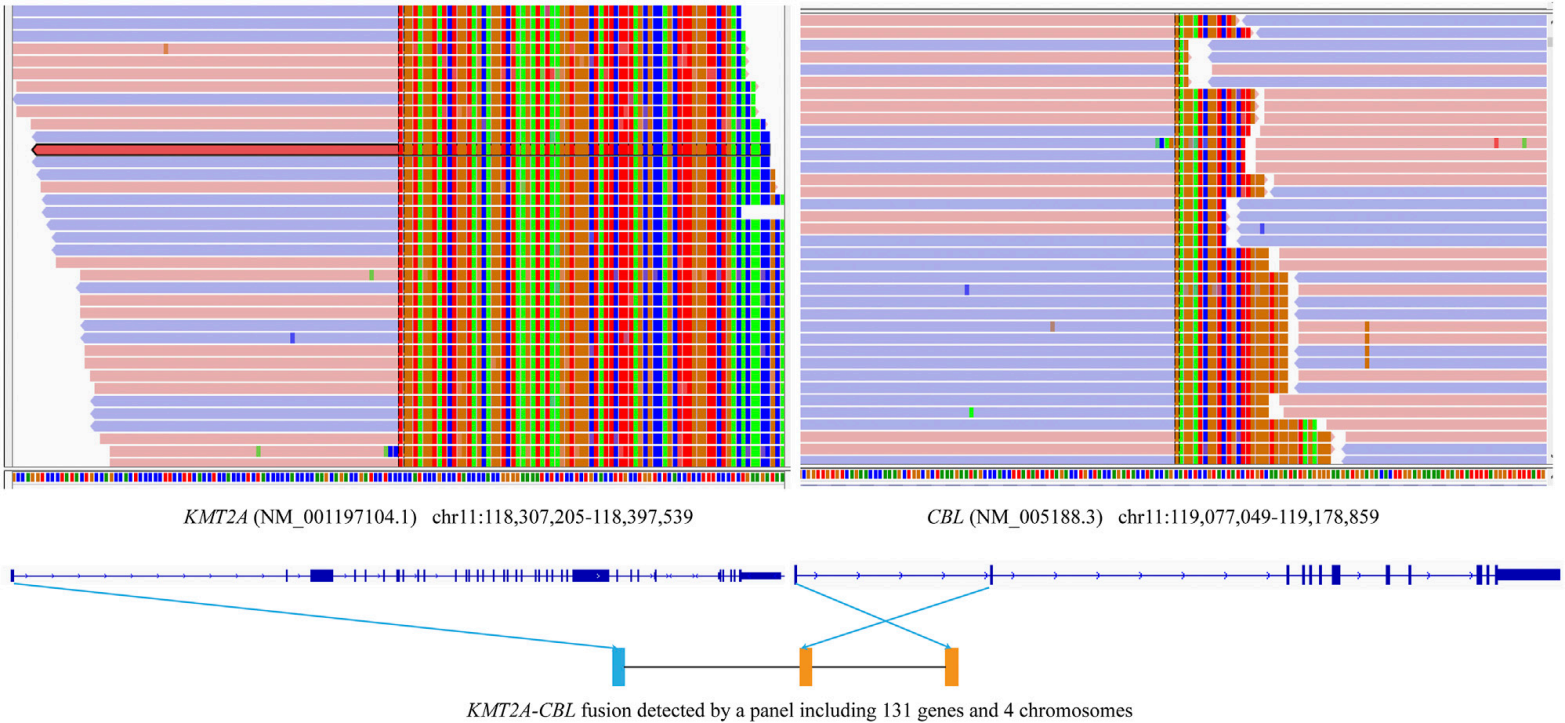
Schematic diagrams of the novel *KMT2A-CBL* fusion. The *KMT2A-CBL* fusion between *KMT2A* exon 1 and *CBL* exon 2 in the tumor of Case #3 using a panel of 131 genes and 4 chromosomes.

*ATRX* is rarely mutated in adults with primary high-grade gliomas, but is more frequent in younger adults with lower-grade gliomas [17]. In our report, *ATRX* mutation was identified in Case #1 who was a young adult with lower-grade gliomas. There are studies suggesting *ATRX* mutations are associated with better prognosis in *IDH*-mutant, low-grade glioma patients without 1p/19q co-deletion [18, 19]. Although no chromosome 1p/19q deletion was found in this report, multiple specific chromosomal alterations were identified in three lower-grade gliomas, including on chromosome 1q, 4q, 5q, 7p, 10q, 11q, 12q, 13q deletions, and more. A previous study showed that astrocytomas progressed rapidly to glioblastoma, with short survival intervals and increased frequencies of 9p, 10q, and 13q deletions [20]. Additionally, the *KMT2A-CBL* fusion previously reported in acute leukemia was first identified in brainstem gliomas. The breakpoints of the *KMT2A* and *CBL* genes in Case #3 were different from those in acute leukemia (Figure 2), which should be validated by other methods like RNA-seq.

## Conclusion

These typical cases suggest that adult *IDH1-*mutant brainstem gliomas have different clinicopathological and genetic characteristics, some of which may be associated with poor prognosis. These findings demonstrate that the specific characteristics of *IDH*-mutant brainstem gliomas should be considered in diagnostic workflows to make therapeutic regimens and improve the prognosis.

## Data Availability

The raw data supporting the conclusion of this article will be made available by the authors, without undue reservation.
