# Supplementary material for: Case Report: Clinicopathological and Genetic Features of IDH-Mutant Brainstem Glioma in Adults: Report of Five Cases
Source: Pathol Oncol Res. 2022 Aug 4;28:1610408. doi: 10.3389/pore.2022.1610408 (PMC9385964; doi:10.3389/pore.2022.1610408)
Supplement: Supplementary file 1 [file Table1.docx]

**Table S1. The SNV, CNV and fusion information of the five adults with brainstem gliomas**

| Case | SNV | | | | | | | | CNV | | Fusion |
| --- | --- | --- | --- | --- | --- | --- | --- | --- | --- | --- | --- |
|  | Chromosome | Position | Allele depth | Depth | VAF | Gene | HGVS_p | Gene | | Copy number |  |
| #1 | chr17 | 7579424 | 41 | 842 | 0.0487 | *TP53* | p.A88Pfs*60 | *CDK4* | | 3.18 | No |
|  | chrX | 76813031 | 35 | 495 | 0.0707 | *ATRX* | p.R2197H | *IRS2* | | 4.23 |  |
|  | chr2 | 209113112 | 637 | 2062 | 0.3089 | *IDH1* | p.R132H |  | |  |  |
| #2 | chr12 | 25398284 | 51 | 2234 | 0.0228 | *KRAS* | p.G12V | *H3F3A* | | 6.43 | No |
|  | chr17 | 7577501 | 1288 | 2159 | 0.5966 | *TP53* | p.S261Vfs*84 |  | |  |  |
|  | chr2 | 209113112 | 688 | 2343 | 0.2936 | *IDH1* | p.R132H |  | |  |  |
| #3 | chr17 | 7577568 | 1278 | 1726 | 0.7404 | *TP53* | p.C238F | *PTEN* | | 1.3 | KMT2A(Exon1)-CBL(Exon2-1) |
|  | chr2 | 209113112 | 671 | 1687 | 0.3977 | *IDH1* | p.R132H |  | |  |  |
| #4 | chr17 | 7577138 | 1065 | 1188 | 0.8965 | *TP53* | p.R267P | CDK6 | | 3.92 | No |
|  | chr1 | 226252135 | 19 | 622 | 0.0305 | *HIST1H3B* | p.K28M |  | |  |  |
|  | chr2 | 209113112 | 517 | 1334 | 0.3876 | *IDH1* | p.R132H |  | |  |  |
| #5 | chr13 | 32937526 | 30 | 87 | 0.3448 | *BRCA2* | p.K2729N | CDK4 | | 3.83 | No |
|  | chr17 | 7577120 | 225 | 261 | 0.8621 | *TP53* | p.R273H |  | |  |  |
|  | chr2 | 209113113 | 50 | 123 | 0.4065 | *IDH1* | p.R132G |  | |  |  |

**Abbreviations:** SNV, single-nucleotide variants; CNV, copy number variants; VAF, variant allele fraction; HGVS, Human Genome Variation Society.
