# Supplementary figures and images for: Case Report: Clinicopathological and Genetic Features of IDH-Mutant Brainstem Glioma in Adults: Report of Five Cases
Source: Pathol Oncol Res. 2022 Aug 4;28:1610408. doi: 10.3389/pore.2022.1610408 (PMC9385964; doi:10.3389/pore.2022.1610408)

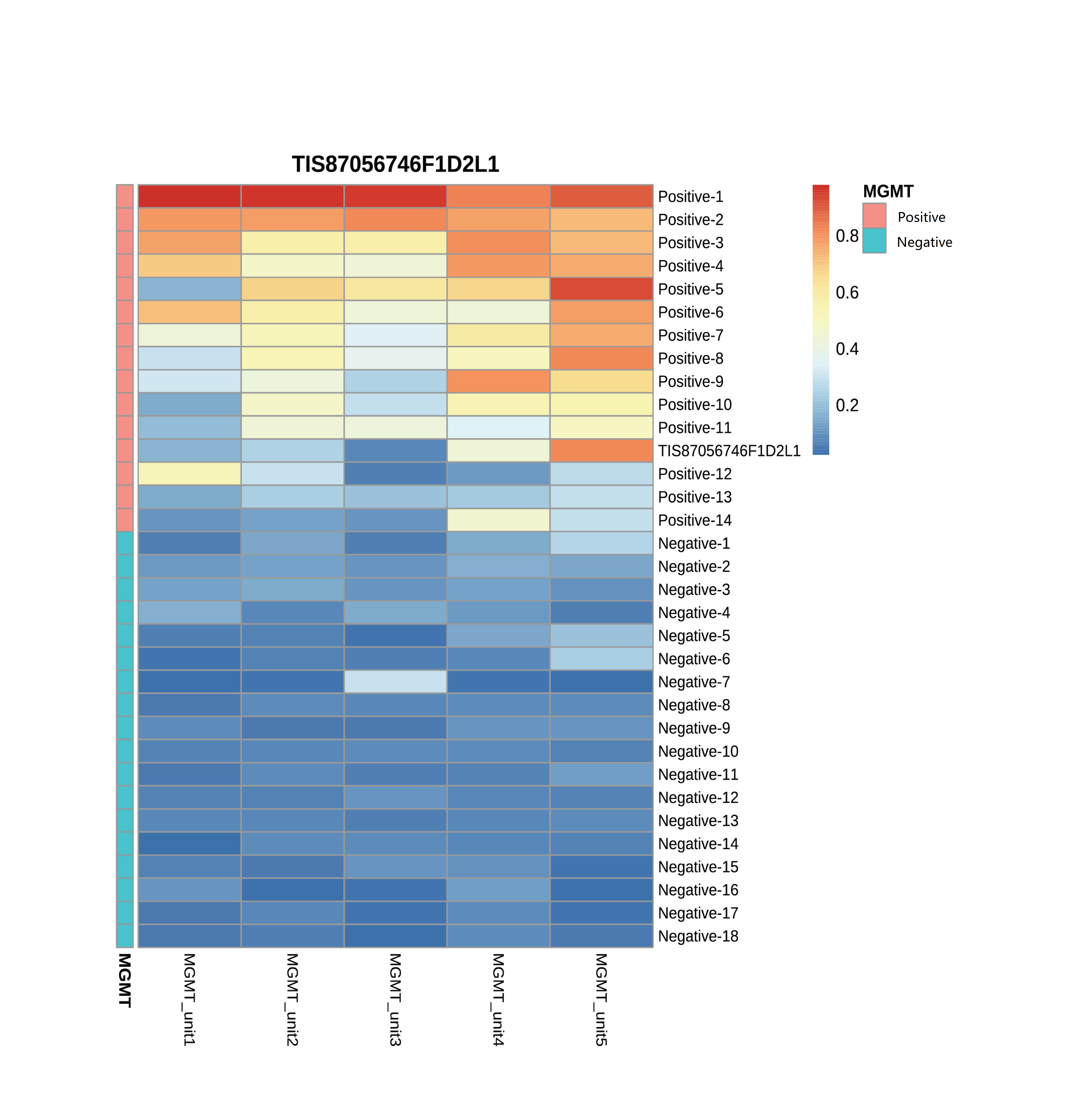

Supplement: Supplementary file 2 [file Image1.TIF]
