# Supplementary material for: Case Report: Clinicopathological and Genetic Features of IDH-Mutant Brainstem Glioma in Adults: Report of Five Cases
Source: Pathol Oncol Res. 2022 Aug 4;28:1610408. doi: 10.3389/pore.2022.1610408 (PMC9385964; doi:10.3389/pore.2022.1610408)
Supplement: Supplementary file 3 [file Table2.docx]

**Table S2. Targeted next-generation sequencing using a panel of 131 genes and 4 chromosomes**

| *ACVR1* | *AKT1* | *AKT2* | ****ALK*** | *APC* | *AR* | *ARAF* | *ARID2* | *ATM* |
| --- | --- | --- | --- | --- | --- | --- | --- | --- |
| *ATRX* | *B2M* | **BCL2L11* | *BCOR* | ****BRAF*** | *BRCA1* | *BRCA2* | *CBL* | *CCND2* |
| *CDK4* | *CDK6* | *CDKN2A* | *CDKN2B* | *CDKN2C* | *CHEK2* | *CIC* | *CTNNB1* | *DAXX* |
| *DDR2* | *DDX3X* | *DICER* | *DNMT3A* | *EGFR* | *#EPCAM* | *ERBB2* | *ERBB3* | *ERBB4* |
| *ESR1* | *FAT1* | *FBXW7* | *FGF4* | *FGFR1* | *FGFR2* | ****FGFR3*** | *FGFR4* | *FLT3* |
| *FLT4* | *FUBP1* | *GNA11* | *GNAQ* | *GNAS* | *H3F3A* | *HDAC4* | *HIST1H3B* | *HIST1H3C* |
| *HMCN1* | *HNF1A* | *HRAS* | *IDH1* | *IDH2* | *IRS2* | *JAK1* | *JAK2* | *KDM5A* |
| *KIT* | *KLF4* | *KRAS* | *MAP2K1* | *MAPK1* | *MDM2* | *MDM4* | *MEN1* | ***#*MET*** |
| *MLH1* | *MPL* | *MSH2* | *MSH6* | *MTOR* | ****MYB*** | *MYC* | *MYCN* | ****NAB2*** |
| *NF1* | *NF2* | *NOTCH1* | *NR3C1* | *NRAS* | ****NTRK1*** | ****NTRK2*** | ****NTRK3*** | *PDGFRA* |
| *PDGFRB* | *PIK3CA* | *PIK3CB* | *PIK3R1* | *PLCG1* | *PMS2* | *POLE* | *POLR2A* | *PPM1D* |
| *PTCH1* | *PTEN* | *PTPN11* | *RAF1* | *RB1* | ****RELA*** | ****RET*** | *RGPD3* | *RICTOR* |
| ****ROS1*** | *SDHA* | *SETD2* | *SMAD4* | *SMARCA4* | *SMARCB1* | *SMARCE1* | *SMO* | *SRC* |
| *STAG2* | ****STAT6*** | *#TERT* | *TP53* | *TRAF7* | *TSC1* | *TSC2* | *USP8* | *VEGFA* |
| *VEGFB* | *VEGFR1* | *KDR* | *VHL* | ****YAP1*** | ***chr***： ***1P*** | ***chr***： ***19Q*** | ***chr***： ***7*** | ***chr***： ***10*** |

**Notes:** Single nucleotide variants (SNVs), insertion/deletion and copy number variation (CNVs) were analyzed in genes marked black; Gene fusion, insertion/deletion, CNVs were analyzed in genes marked red; Chromosomal copy number alterations were identified in genes marked blue. *Cover all exons, and all introns of some genes; #Cover the promoter region.
